# Electrical stimulation enhances mitochondrial trafficking as a neuroprotective mechanism against chemotherapy-induced peripheral neuropathy

**DOI:** 10.1016/j.isci.2024.109052

**Published:** 2024-01-30

**Authors:** Bayne Albin, Prashant Adhikari, Arjun Prasad Tiwari, Khayzaran Qubbaj, In Hong Yang

**Affiliations:** 1 Center for Biomedical Engineering and Science, Department of Mechanical Engineering and Engineering Science, University of North Carolina at Charlotte, Charlotte, NC 28223, USA

**Keywords:** Biochemistry, Molecular biology, Neuroscience

## Abstract

Electrical stimulation (ESTIM) has shown to be an effective symptomatic treatment to treat pain associated with peripheral nerve damage. However, the neuroprotective mechanism of ESTIM on peripheral neuropathies is still unknown. In this study, we identified that ESTIM has the ability to enhance mitochondrial trafficking as a neuroprotective mechanism against chemotherapy-induced peripheral neuropathies (CIPNs). CIPN is a debilitating and painful sequalae of anti-cancer chemotherapy treatment which results in degeneration of peripheral nerves. Mitochondrial dynamics were analyzed within axons in response to two different antineoplastic mechanisms by chemotherapy drug treatments paclitaxel and oxaliplatin *in vitro*. Mitochondrial trafficking response to chemotherapy drug treatment was observed to decrease in conjunction with degeneration of distal axons. Using low-frequency ESTIM, we observed enhanced mitochondrial trafficking to be a neuroprotective mechanism against CIPN. This study confirms ESTIM enhances regeneration of peripheral nerves by increased mitochondrial trafficking.

## Highlights

• Pulsed electrical Stimulation has the ability to increase axonal growth rates at low frequencies of 10 Hz, 100 Hz, and 1,000 Hz
• Electrical stimulation has neuroprotective abilities against common chemotherapy-induced peripheral neuropathies (CIPNs)
• Pulsed electrical stimulation has the ability to increase anterograde mitochondrial trafficking rates at low frequencies of 10 Hz, 100 Hz, and 1,000 Hz
• A potential mechanism behind electrical stimulation-mediated neuroprotection is increased mitochondrial trafficking

## Graphical abstract

**Figure:**
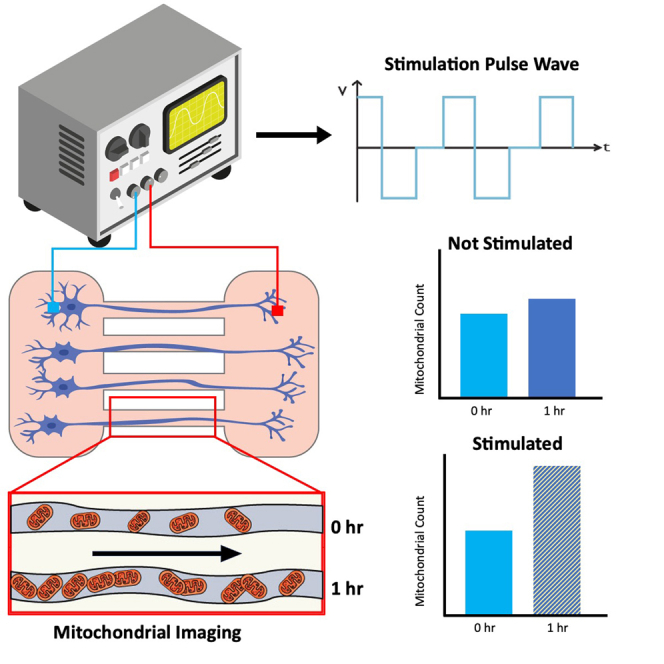

## Introduction

The use of electrical stimulation (ESTIM) on peripheral nerves has been well studied since 1967, when Wall and Sweet reported on the use of ESTIM for pain relief for the first time.^1^ For the past 50 years, neuromodulation has been implemented in many ways to reduce pain, or as a treatment method for neurological diseases and conditions.^2^ Although peripheral nerves have significant regenerative capabilities compared to other cell types, functional recovery is proven to be poor. ESTIM is currently available to the public as a treatment strategy to mitigate pain seen in the form of a transcutaneous electrical nerve stimulator (TENS).^3^ ESTIM is approved for a variety of clinical situations, including physical therapy, spinal cord injuries, neuro-prosthesis, mental health, and neuropathy among others.^4^^,^^5^^,^^6^ Many researchers believe that the use of ESTIM on peripheral nerve injuries may be a key therapy for peripheral nerve regeneration in clinical settings.^7^ Pulsed ESTIM has shown to be an effective treatment strategy for peripheral injury conditions.^8^^,^^9^ Previous studies have shown that using a low-frequency (>100 Hz) pulsed biphasic stimulation for 1 h per day is able to increase sensory nerve regeneration and myelination. Low-frequency stimulation is also considered to be optimal conditions for nerve growth and protection;^7^^,^^10^^,^^11^^,^^12^ however, it is important to identify a larger range of frequencies to fully understand the effect of ESTIM against chemotherapy-induced peripheral neuropathy (CIPN) by paclitaxel (PTX) and oxaliplatin (L-OHP).^7^^,^^13^^,^^14^ Although ESTIM has been a well-researched phenomenon, the underlying mechanism regarding peripheral nerve regeneration is poorly understood.

Mitochondrial population and trafficking are significant indicators of neuronal health.^15^ Low population and trafficking of mitochondria along the axons can be directly related to low energy production, which in turn can lead to peripheral nerve degeneration and cell death.^16^ Energy demands within the neuron change dependently on location, circumstance, and injury.^17^ For example, the growth cone and synapse require significant energy resources in order to deliver signals to target locations.^18^ Due to this high, dynamic energy demand, mitochondria are needed in different locations as well as in different densities to supply energy in the form of ATP on demand.^19^ Mitochondrial trafficking and dynamic responses help maintain homeostasis regarding energy demands. Neuronal activity has been found to affect mitochondrial trafficking responses, where increased signaling and neuronal activity increase mitochondrial trafficking in localized regions of the neuron.^20^ The dynamic nature of energy distribution by mitochondrial trafficking is an important mechanism to understand, due to increased energy demands in a disease state such as CIPN.^21^^,^^22^ The effect of specific drugs and treatment methods on mitochondrial transport can help provide an indication on the targeting mechanism for such treatment strategies. Using this information, better methods can be developed to alter subcellular trafficking for neuroprotection. Using imaging techniques, it is possible to determine the trafficking response and communication between transport mechanisms.

CIPN is a debilitating health condition associated with common chemotherapy drugs.^23^ CIPN is a painful side effect of chemotherapy treatment which affects 67% of all cancer patients undergoing chemotherapy treatment.^24^ Chemotherapy is an effective treatment method targeting the cancer cell division process, to eliminate cancer from the body. However, there are many adverse side effects as a result of chemotherapy treatment, including peripheral neuropathy. Currently, there are no treatment methods for peripheral neuropathy other than symptom mitigation.^25^ CIPN is the most common dose-limiting factor during chemotherapy treatment, oftentimes leading to suboptimal conditions to fight cancer, and even termination of treatment in extreme cases. Using this knowledge ESTIM is shown to be an effective treatment option against common chemotherapy drugs, L-OHP and PTX. L-OHP and PTX were chosen due to their common application in chemotherapy treatment which results in CIPN, as well as to compare against different targeting mechanisms.

PTX is known as an effective agent in disrupting the cell division cycle of cancer cells by targeting the microtubules associated with mitosis.^26^ PTX is primarily used against breast, lung, and ovarian cancer types.^27^ Due to the microtubule targeting effect of PTX, it can be assumed that PTX is directly related to axonal trafficking. The effect of ESTIM on PTX-induced peripheral neuropathy (PIPN) was studied based on our recent study identifying fluocinolone acetonide (FA) as a neuroprotective agent, which enhances mitochondrial trafficking leading to neuroprotection against common CIPNs.^28^ To fully understand the mechanism of neuroprotection by ESTIM, another drug with different targeting mechanism is needed for comparison. L-OHP is a third-generation platinum-based antineoplastic chemotherapy drug primarily used to target colorectal and stage III colon cancer.^29^ L-OHP targets the DNA replication cycle by binding to DNA strands and disrupting the transcription process within cancer cells.^30^ DNA damage associated with L-OHP treatment is crucial to anti-cancer treatment as it can disrupt tumor development. CIPN is the leading side effect associated with L-OHP treatment, which can cause major discomfort and pain leading to dosage reductions for patients.^31^ The specific mechanisms of toxicity in CIPN for L-OHP are still unknown; however, it is speculated that transporter-mediated uptake of L-OHP triggers pathophysiological changes seen as disrupted cell signaling cascades, mitochondrial disfunction, and oxidative stress in dorsal root ganglion neurons (DRGs). L-OHP also has been noted to have a higher toxicity effect on mitochondria within targeted cells.^32^^,^^33^ Using this information, we seek to determine if ESTIM is capable of protecting mitochondrial transport even when directly targeted by L-OHP. Therefore, L-OHP and PTX were chosen to determine if ESTIM was effective at enhancing mitochondrial trafficking and subsequent neuroprotection. The two different mechanisms of action for these drugs are the most commonly used types of chemo drugs, and it is important to understand how ESTIM affects both mechanisms. In order to analyze the effect of ESTIM-mediated neuroprotection against PTX- and L-OHP-induced peripheral neuropathy, axon length responses were measured with varying pulsed ESTIM frequencies (10 Hz–1 MHz). DRGs were similarly stimulated using a compartmentalized cell culture chamber to determine the effect of the frequency range on mitochondrial trafficking. Frequencies which showed positive axon length and mitochondrial responses were used in conjunction with PTX and L-OHP to determine the neuroprotective effect against CIPN. This is the first time that ESTIM is shown to have the ability to enhance mitochondrial trafficking in axons that guarantees neuroprotection against CIPN.

## Results

### Experimental setup

**Figure 1:**
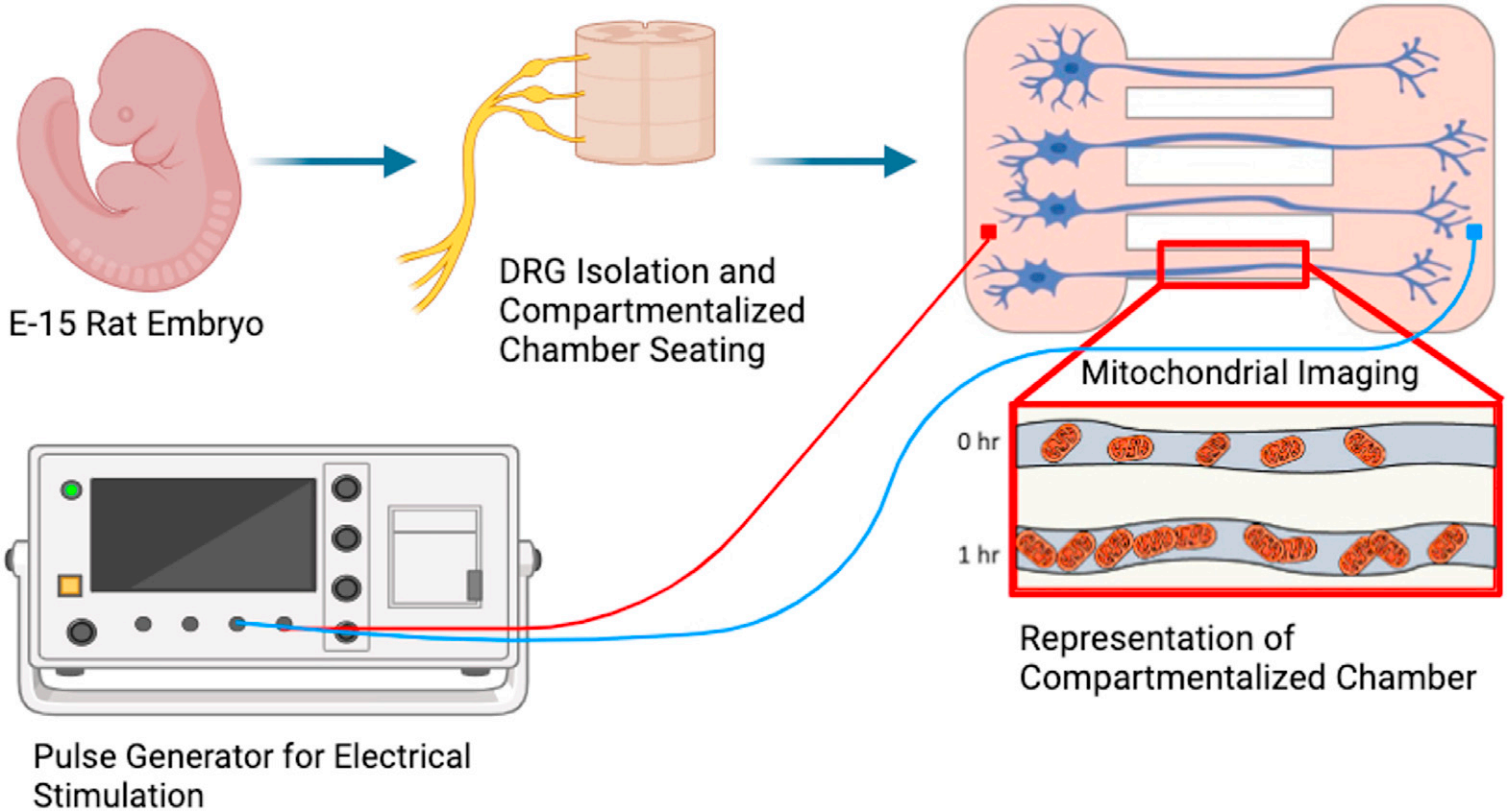
Experimental schematic E-15 Sprague-Dawley rat embryonic dorsal root ganglion (DRG) isolation and enzymatic disassociation. Processed DRGs plated into the cell body chamber of the compartmentalized chamber system. Cells are allowed to grow for 3–5 days until they grow through the channels to the axonal compartment, where chemotherapy drugs and dyes are administered. Electrical stimulation was performed for 1 h per day using a constant 3 V power supply to generate a biphasic rectangular pulse lasting 0.5 s every 2 s with a pulse width of 0.2 ms. Imaging of axons was completed in the axonal compartment, while mitochondrial trafficking imaging was performed within the parallel microchannels. Created with BioRender.com.

Figure 1 illustrates the experimental schematic for the outlined ESTIM experiments. DRGs were isolated and processed from E-15 pregnant rats and cultured on a custom 12-well compartmentalized culture plate seen in Figure 1. Chemotherapy drugs and fluorescent dyes were treated in culture chambers and incubated for 1 h prior to ESTIM. ESTIM was performed by sending biphasic square pulse wave signals from a Siglent waveform generator to platinum electrodes attached to the lid of the culture device. Imaging using a Leica-DMi8 fluorescent microscope was performed before and after 1 h of stimulation to determine axon length or mitochondria trafficking response.

### Global stimulation frequency response

**Figure 2:**
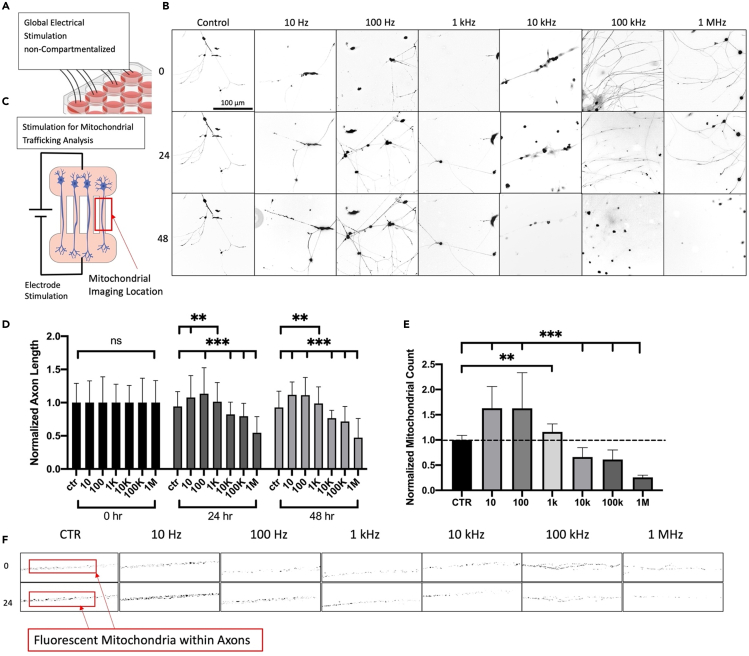
Pulsed electrical stimulation enhances axon growth and mitochondrial trafficking Schematic for global DRG stimulation for axon length measurement analysis (A). The axonal response after stimulation for 0, 24, and 48 h, for 10Hz, 100Hz, 1 kHz, 10 hHz, 100 kHz, and 1 MHz showed that low frequency (10 Hz, 100 Hz, and 1 kHz) proved to enhance axon length measurements at each timestamp (B). Mitochondrial imaging was performed in a compartmentalized microfluidic chamber to identify the immediate trafficking response to each frequency (C). Quantification of normalized axon length measurements were used to compare against the initial 0 h length (D). Mitochondria were shown to increase between the 0 h and 1 h stimulation for low frequency stimulation parameters (E). Mitochondria were imaged before and after 1 h stimulation to identify the trafficking enhancement by ESTIM (F). It was proven that 100 Hz stimulation enhanced mitochondrial trafficking the most compared to control samples (E). Data are shown by mean ± SD. ∗∗∗p < 0.001, ∗∗p < 0.01, ∗p < 0.05 as determined by two-tailed t test (E), Scale bar is 100 μm unless otherwise stated.

To determine the effect of each frequency on axon length, DRGs were seated in a non-compartmentalized sterile 24-well glass-bottom cell culture plate shown in Figure 2A. DRGs were cultured and allowed to grow for 24 h prior to stimulation. 1 h stimulation at 10 Hz, 100 Hz, 1 kHz, 100 kHz, and 1 MHz was induced to the cells once per day for 3 days as shown in Figures 2B and 2C. Cells were treated with Calcien acetoxymethyl ester (AM) and imaged before and after stimulation using a fluorescent microscope. Fluorescent images were processed using Fiji-ImageJ to measure axon length at each corresponding frequency and timestamp. As seen in Figures 2B and 2C, the frequency response was characterized, where low frequencies (>1 kHz) enhanced axon growth, whereas high frequencies (<1 kHz) had a detrimental effect on axon length. Over the three-day stimulation period, 10 Hz accelerated growth by 9.71% ± 0.26%, 100 Hz accelerated growth by 12.28% ± 0.33%, and 1 kHz accelerated growth by 0.07% ± 0.27%, whereas 100 kHz inhibited growth by 24.43% ± 0.21% and 1 MHz inhibited growth by 49.03% ± 0.27%. Based on these values, optimal frequencies were chosen when preforming the experiment alongside chemotherapy drugs to characterize the neuroprotective effect of each frequency. Seen in Figure 2B, representative figures for 0, 24, and 48 h when exposed to the 5 different frequencies are shown. Graphical representation of axon length as a comparison to the control can be seen from Figure 2C. After 24 and 48 h, 100 Hz had the highest growth rate. These results are found to be consistent with current research, showing low-frequency stimulation to have the largest positive impact on axon growth.

Fluorescent imaging of mitochondria was performed for each frequency to characterize the trafficking response to the full frequency range (10 Hz–1 MHz). In order to properly measure the frequency response on mitochondrial trafficking, DRGs were seated into the polydimethylsiloxane (PDMS)-based microchannel device and allowed to grow for 3–5 days in order to ensure proper growth through the microchannels. MitoView Green dye was then added to the cell body chamber and incubated in correspondence with the manufacturer’s protocol. Fluorescent imaging of mitochondria before and after stimulation at each frequency can be seen in Figure 2F. The specific timestamps in Figure 2C were chosen to measure the direct impact of each frequency on mitochondrial count after 1 h stimulation. Variables, such as dye degradation, media changes, and light exposure, had the potential to skew data if recorded over an extended period of time; therefore, mitochondrial images were collected directly before and after 1 h stimulation at each frequency. A microfluidic compartmentalized cell culture system was used to properly stimulate and identify mitochondrial movement within the axons. Using a parallel array of microchannels, DRGs were compartmentalized in order to fully isolate the cell bodies from the axons. By doing this, axons are aligned perfectly within the microchannels, enabling us to image and count mitochondrial population at each timestamp. MitoView Green dye was placed into the cell body chamber of the compartmentalized system, while neurobasal media were added in higher volume to the axonal chamber, inducing hydrostatic pressure to prevent excess dye diffusion. Cell body dye administration further helped show anterograde movement of mitochondria to focus on the change in energy demand at the site of injury during CIPN. Using these data, we were able to understand how mitochondria were affected by the initial stimulation. After 1 h stimulation, mitochondria population increased by 62.79% ± 0.43% for 10 Hz, 62.59% ± 0.71% for 100 Hz, and 15.96% ± 0.16% for 1 kHz but decreased by 38.90% ± 0.19% for 100 kHz and 74.28% ± 0.04% for 1 MHz. All values were normalized according to the 0-h control count to provide a proper increase/decrease ratio. Values above “1.0” represent an increase in mitochondrial number, whereas values below “1.0” represent a decrease. The trends seen in the results for mitochondrial population match those with axon length measurements leading us to believe ESTIM directly modulates mitochondrial trafficking and other subcellular axonal trafficking mechanics to increase axonal growth.

### Global ESTIM as a neuroprotective method against CIPN

**Figure 3:**
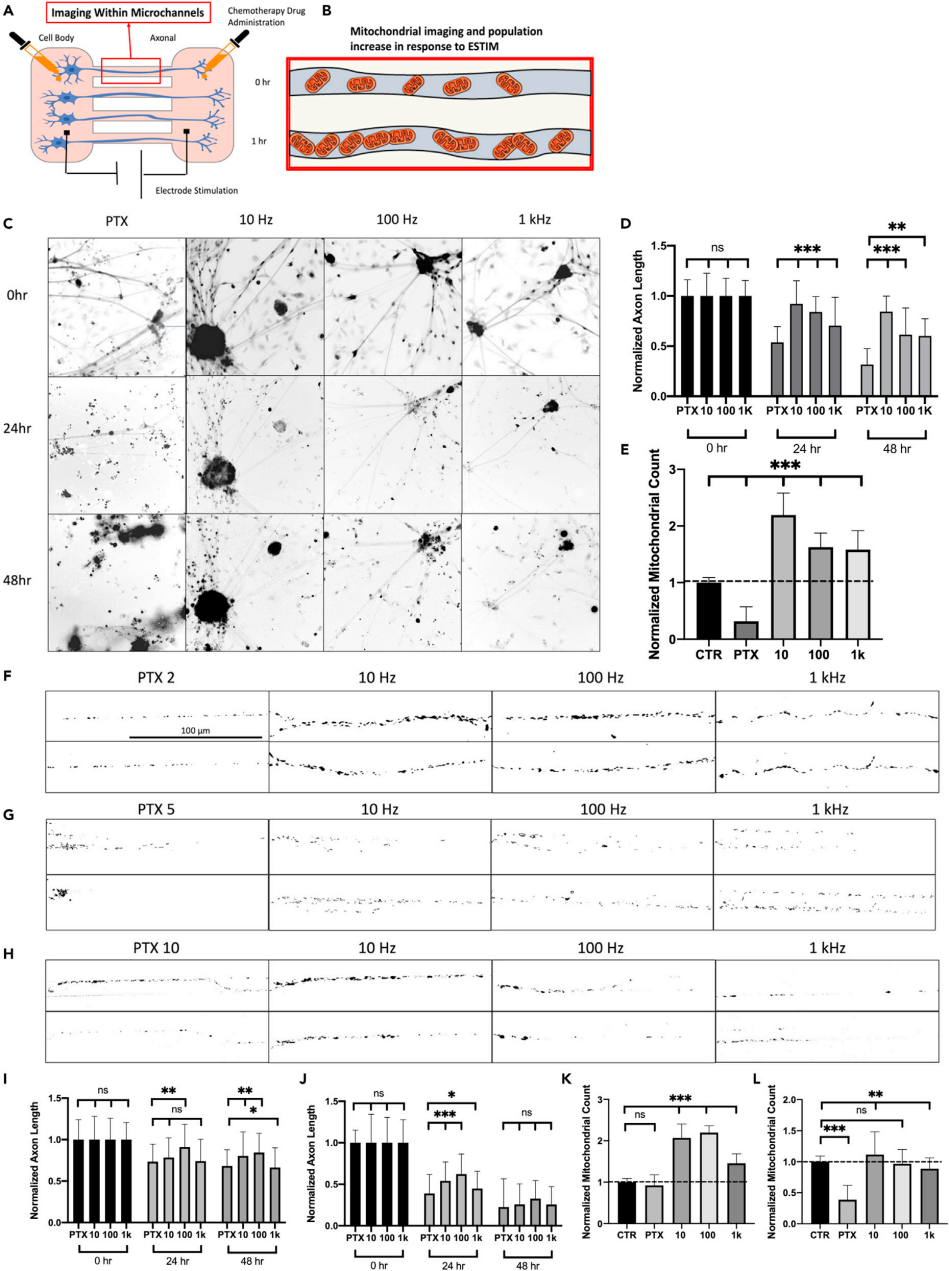
Electrical stimulation enhances mitochondrial trafficking as a neuroprotective mechanism against PTX Global stimulation setup using 10 Hz, 100 Hz, and 1 kHz (A). (B) Schematic of mitochondrial dynamics during ESTIM. PTX-treated samples were stimulated for 1 h per day using the same parameters as frequency response (C). Quantification of neuroprotection by ESTIM against PTX 5 μM-induced peripheral degeneration (D). (E) Quantification of normalized mitochondrial count as compared to control samples during ESTIM for PTX 5 μM. Quantification of neuroprotection by ESTIM against PTX 2 μM- and 10 μM-induced degeneration, respectively (I, J). Mitochondrial imaging was performed using the compartmentalized chamber system where imaging was preformed within microchannels after axons grew through (A). Image analysis of mitochondrial trafficking effect of 1-h ESTIM after PTX treatment using 2 μM, 5 μM, and 10 μM (F‒H). Quantification of mitochondrial trafficking effect by ESIM against PTX 2 μM and 10 μM (K, L). Data are shown by mean ± SD. ∗∗∗p < 0.001, ∗∗p < 0.01, ∗p < 0.05 as determined by two-tailed t test, Scale bar is 100 μm unless otherwise stated.

**Figure 4:**
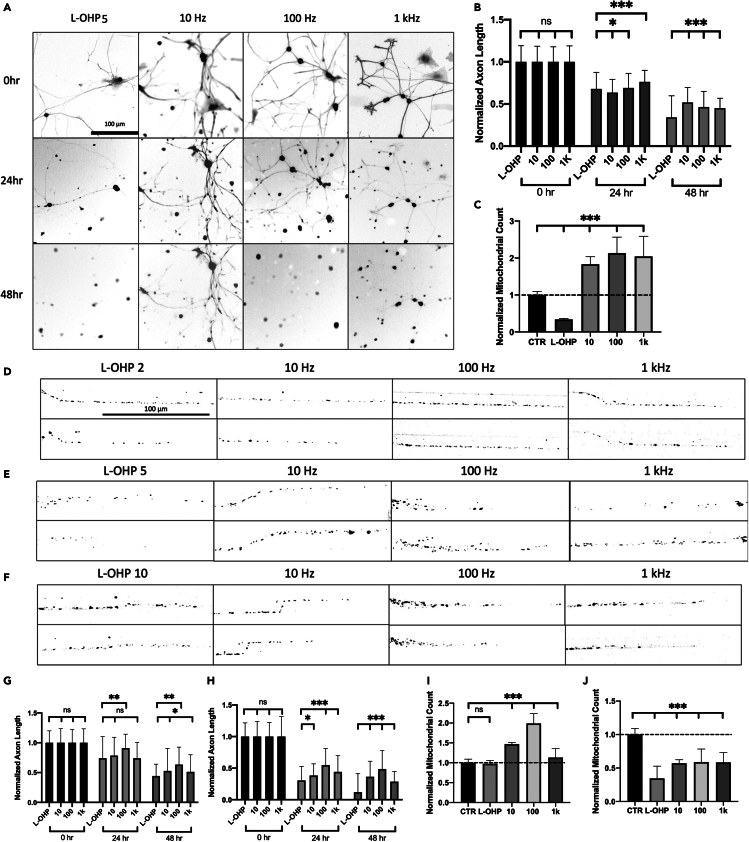
Electrical stimulation enhances mitochondrial trafficking as a neuroprotective mechanism against L-OHP L-OHP-treated samples were stimulated for 1 h per day using the same parameters as frequency response (A). Quantification of neuroprotection by ESTIM against L-OHP 5 μM-induced peripheral degeneration (B). (C) Quanification of normalized mitochondrial trafficking effect during ESTIM at L-OHP 5 μM. Quantification of neuroprotection by ESTIM against L-OHP 2 μM- and 10 μM-induced degeneration respectively (G, H). Image analysis of mitochondrial trafficking effect of 1-h ESTIM after L-OHP treatment using 2 μM, 5 μM, and 10 μM (D‒F). Quantification of mitochondrial trafficking effect by ESIM against L-OHP 2 μM and 10 μM (I, J). Data are shown by mean ± SD. ∗∗∗p < 0.001, ∗∗p < 0.01, ∗p < 0.05 as determined by two-tailed t test, Scale bar is 100 μm unless otherwise stated.

The same global stimulation experimental setup was used to identify the neuroprotective effect of ESTIM against CIPN by PTX and L-OHP shown in Figures 3 and 4, respectively. Optimal concentrations for PTX and L-OHP with *in vivo* experimentation range from 5 to 20 mg/kg and 5–25 mg/kg, respectively.^34^^,^^35^ Based on *in vivo* settings, *in vitro* concentrations were chosen which highlighted proper dying-back neuropathy. PTX and L-OHP were tested using concentrations 2 μM, 5 μM, and 10 μM, where axon length and mitochondrial response were characterized. For each concentration, neurons were stimulated using 10 Hz, 100 Hz, and 1 kHz. The optimal concentration of PTX and L-OHP was determined to be 5 μM, where PTX and L-OHP showed degradation of axons without inducing sudden cell death.^22^^,^^36^^,^^37^ Based on the toxicity response of PTX and L-OHP, concentrations of 5 μM are used for additional results. Axon length results showed that PTX and L-OHP at 5 μM had an average degradation of 67.85% and 65.97% after 3-day exposure compared to control, respectively. For PTX, ESTIM was found to inhibit degradation, where 10 Hz showed 15.55%, 100 Hz, 38.57%, and 1 kHz, 39.75% degradation, showing a decrease in PTX degradability where 10 Hz had the highest protection. Alternatively, ESTIM was found to be neuroprotective against L-OHP treatment where 10 Hz showed 48.12%, 100 Hz, 53.74%, and 1 kHz, 54.95% degradation. Although PTX and L-OHP had different targeting mechanisms, ESTIM was found to have a neuroprotective effect on both the drugs. This result leads us to conclude that the underlying mechanism of neuroprotection by ESTIM could be similar if not the same.

### Focal stimulation as a neuroprotective method against CIPN

**Figure 5:**
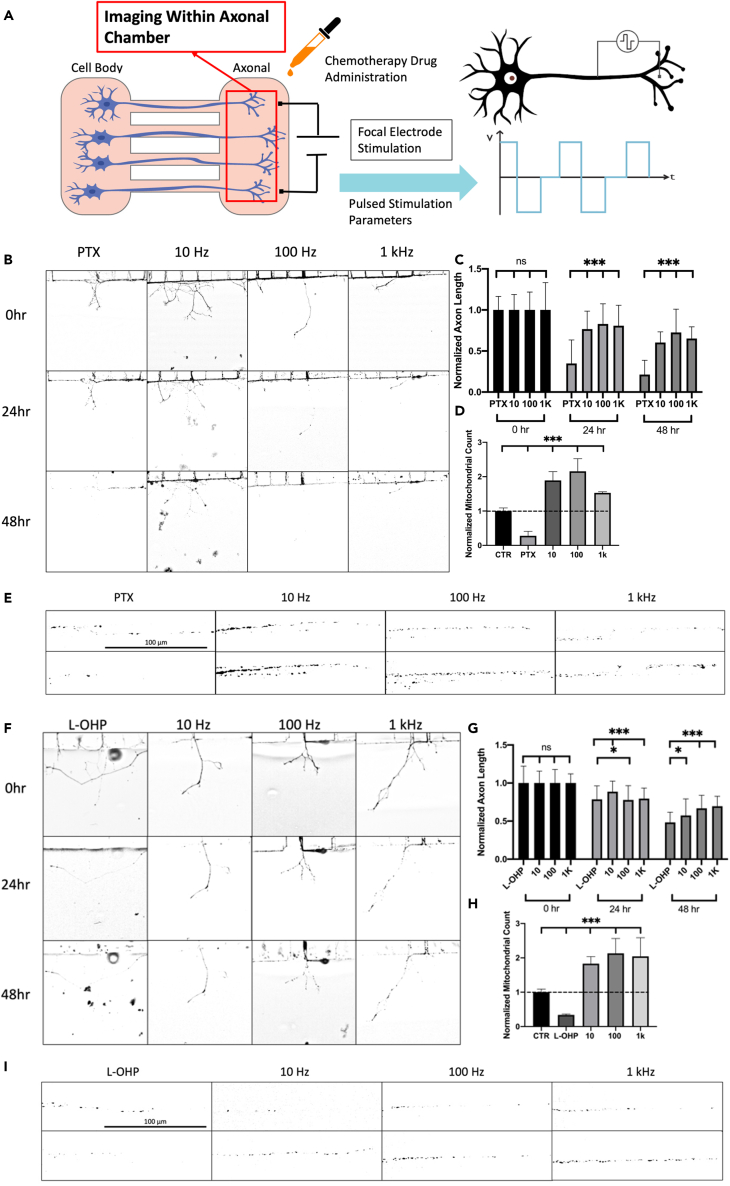
Focal pulsed stimulation on DRGs using the microfluidic compartmentalized cell culture chamber Global drug treatment was administered using PTX and L-OHP with optimal concentrations (A). Axon length measurements were collected and quantified in response to PTX treatment and ESTIM (B, C). Mitochondrial imaging was performed before and after stimulation at each frequency (D, E). Axon length measurements were collected and quantified in response to L-OHP treatment and ESTIM (F, G). Mitochondrial imaging was performed before and after stimulation at each frequency (H, I). Data are shown by mean ± SD. ∗∗∗p < 0.001, ∗∗p < 0.01, ∗p < 0.05 as determined by two-tailed t test, Scale bar is 100 μm unless otherwise stated.

Axonal stimulation was used to identify the neuroprotective effect against PTX- and L-OHP-induced CIPN. It was important to understand any differences between global and focal stimulation due to treatment application of ESTIM against CIPN. Focal vs. global ESTIM can provide important data regarding treatment methodologies in a clinical setting. Focal stimulation of DRGs was performed by placing both electrodes in the axonal compartment. In a previous paper, it was determined that the electric field was localized to the axonal compartment when preforming focal stimulation.^38^ The same parameters and experimental setup were used in order to ensure proper electrical field isolation. Chemotherapy drugs were added in both compartments, mimicking global treatment similar to Figures 2, 3, and 4. ESTIM was performed in the axonal compartment using the same optimized parameters as those of the global stimulation. Focal ESTIM proved to be just as effective as global stimulation for neuroprotection against CIPN seen in Figure 5. Axon length results showed that PTX and L-OHP at 5 μM had an average degradation of 78.69% and 51.66% compared to control, respectively, seen in Figures 5B, 5C, 5F, and 5G. For PTX-treated samples, ESTIM was found to have a neuroprotective effect, where 10 Hz showed only 39.65%, 100 Hz, 27.38%, and 1 kHz, 34.78% degradation, showing a decrease in PIPN effect on DRGs, where 10 Hz had the highest neuroprotection. Alternatively, ESTIM was also found to be neuroprotective against L-OHP treatment where non-stimulated samples showed 51.66% degradation; however, 10 Hz showed 42.58%, 100 Hz, 33.25%, and 1 kHz, 30.48% degradation. Focal stimulation effect in response to PTX and L-OHP can be seen in Figure 5.

### Mitochondrial motility

**Figure 6:**
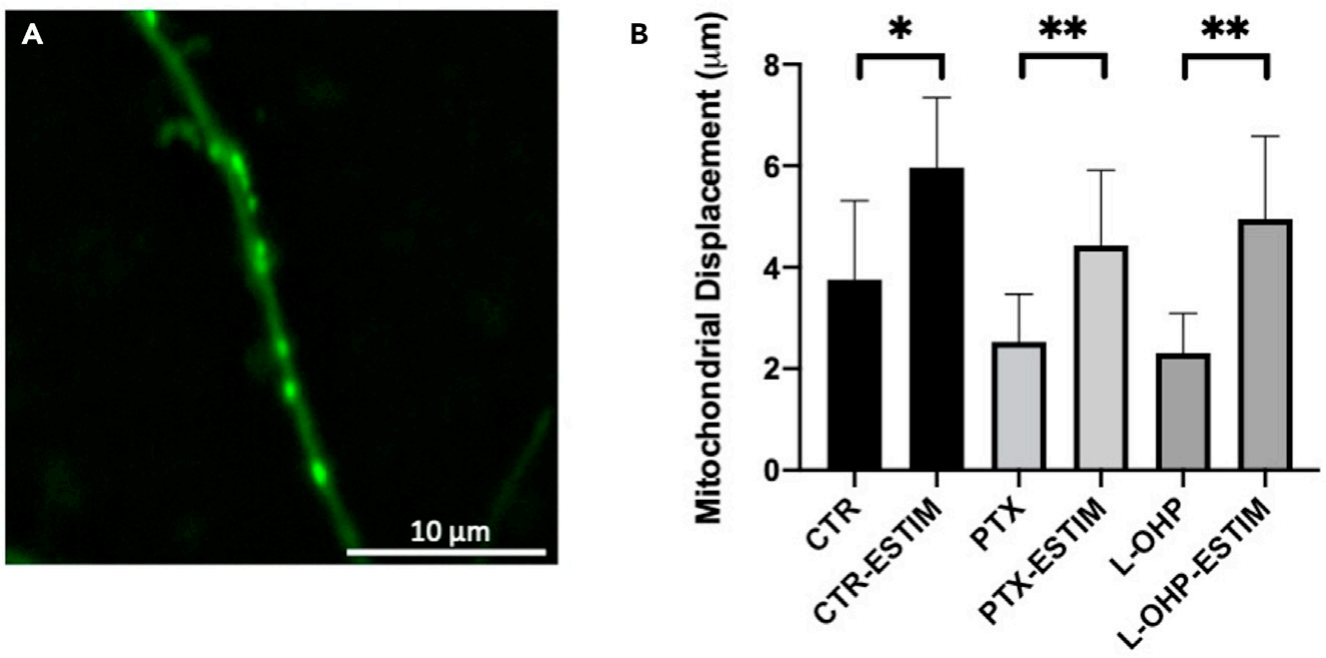
Mitochondrial motility analysis Mitochondrial staining using MitoView Green within axonal section of DRG (A). Mitochondrial velocity with drug-treated samples and 100 Hz ESTIM (B). Data are shown by mean ± SD. ∗∗p < 0.01, ∗p < 0.05 as determined by two-tailed t test.

Figure 6 shows representative time-lapse imaging captured during a 30-min stimulation period. Using a mitochondrial staining approach, displacement of motile mitochondria using time-lapse imaging was determined.^28^ Using MitoView Green dye, mitochondrial displacement was characterized in response to optimal stimulation parameters. DRGs were cultured in a non-compartmentalized glass-bottom culture dish. A non-compartmentalized device was used due to the ease of use, and mitochondrial clarity when stained. Using 100 Hz global ESTIM, displacement of mitochondrial movement was determined in response to PTX and L-OHP treatment. Displacement over the 30-min time period was determined to be 3.76 ± 1.55 μm on average for untreated samples and increased to 5.96 ± 1.38 μm during 100 Hz stimulation. PTX treatment measured displacement to be reduced to 2.53 ± 0.94 μm but increased to 4.43 ± 1.48 μm when stimulated at 100 Hz. Similarly, L-OHP treatment showed an average displacement of 2.31 ± 0.78 μm, increased to 4.95 ± 1.63 μm when stimulated at 100 Hz. All samples were dyed and treated for 1 h prior to time-lapse imaging. Samples were stimulated using the same parameters as those in all other experiments. Based on velocity determination, 100 Hz stimulation was able to accelerate mitochondrial trafficking by 58.36% for untreated, 42.90% for PTX, and 53.36% for L-OHP. Changes in velocity represent significant movement of motile mitochondria in response to the different conditions outlined. Motile mitochondria were analyzed due to their ability to regulate energy demands along the axon.^39^ Based on the results from Figure 6, neuroprotection by ESTIM-enhanced mitochondrial transport can further be assumed.

## Discussion

ESTIM has a great potential as a symptomatic pain management option for chronic pain conditions.^40^ In this study, we determined the neuroprotective effect of ESTIM against CIPN. Prior to determination, optimal frequencies for stimulation were determined. Optimal parameters were used to analyze the axonal response to ESTIM-mediated neuroprotection. ESTIM was found to increase axon length when stimulated at low frequencies; however, it was inhibited at higher frequencies (>1 kHz). Mitochondrial trafficking response was then characterized using the full range of frequencies, where only low frequencies (<1 kHz) showed enhanced trafficking effect. Using the optimal frequencies, PTX- and L-OHP-treated samples were stimulated to determine ESTIM-enhanced neuroprotection and mitochondrial trafficking against CIPN.

This study focused on axonal regeneration and trafficking response to global ESTIM using a frequency range of 10 Hz, 100 Hz, 1 kHz, 10 kHz, 100 kHz, and 1 MHz. Results from the frequency response showed that, over the three-day stimulation period, 10 Hz accelerated axonal growth by 9.71% ± 0.26%, 100 Hz accelerated growth by 12.28% ± 0.33%, and 1 kHz accelerated growth 0.07% ± 0.27%, whereas 10 kHz inhibited growth by 24.43% ± 0.21%, 100 kHz inhibited growth by 24.43% ± 0.21%, and 1 MHz inhibited growth by 49.03% ± 0.27%. As expected, low-frequency (<1 kHz) stimulation increased growth rates of DRGs. Historically, ESTIM has been reported to increase neural activity within neuron cells when exposed to low-frequency stimulation.^41^ The increased neural activity induced by low-frequency stimulation within embryonic DRGs showed increases in axonal growth rates significantly.^42^ Subsequently, mitochondria trafficking was analyzed due to its significance in energy production and overall health of the neuron, as well as its association with neurodegenerative disorders.^43^^,^^44^ Mitochondrial trafficking response to the frequency range was analyzed using fluorescent dyes to take live image of the effect of ESTIM. ESTIM enhanced mitochondrial trafficking by 62.79% ± 0.43% for 10 Hz, 62.59% ± 0.71% for 100 Hz, and 15.96% ± 0.16% for 1 kHz but decreased 33.78% ± 0.18% for 10 kHz, 38.90% ± 0.19% for 100 kHz, and 74.28% ± 0.04% for 1 MHz. Control samples showed mitochondrial population increase of 0.05% ± 0.09% which can be attributed to mitochondrial motility under normal conditions. Although no external stimulation was provided, motile mitochondria are still seen to traffic in an anterograde manner shown in Figure 2 due to changing energy demands.^39^ Due to the increased neural activity induced by ESTIM, we believe total axonal transport was enhanced as shown by the mitochondrial trafficking data. Increased axonal transport is a key component in plasticity development which can be correlated to the increased neural activity by ESTIM.^45^^,^^46^^,^^47^ Enhanced mitochondrial trafficking is likewise heavily associated with increased ATP levels.^48^ Increased ATP by enhanced mitochondrial trafficking is directly correlated with neuroprotection in peripheral nerve injuries.^28^^,^^49^ The findings for axon length and mitochondrial trafficking correlate significantly, as the most effective increase in trafficking and axon length seemed to be 100 Hz. High-frequency stimulation (>1 kHz) showed a decrease in axon length and mitochondrial trafficking. These findings were expected as high-frequency stimulation is a known phenomenon to induce excitotoxicity in neurons.^50^ Due to the negative effect of high-frequency stimulation, further experimentation opted to not use these frequencies. The global stimulation results show significant findings regarding mitochondrial trafficking enhancement, where proper application could result in neuroprotection against CIPN.

In this study we show increased mitochondrial trafficking because of low-frequency ESTIM; however, the underlying mechanism behind this observation is still unclear. Future studies are necessary to determine signaling pathways associated with ESTIM-enhanced mitochondrial trafficking. Herein, we believe one potential mechanism that could be attributed to enhanced trafficking observed in this study is increased brain-derived neurotrophic factor (BDNF) expression.^51^ BDNF is thought to have a direct association with peripheral nerve regeneration, due to its increased expression in injured conditions. BDNF is shown to have rapid upregulation which can be sustained for weeks, strongly suggesting its role in regeneration.^52^^,^^53^ One study suggests that during ESTIM intracellular Ca^2+^ is increased, leading to overexpression of BDNF.^54^^,^^55^ Recently, BDNF is believed to modulate mitochondrial trafficking through downstream activation of protein kinase A.^56^ Further research is needed to correlate enhanced BDNF expression by ESTIM and enhanced mitochondrial trafficking.

Previously we determined that PTX has the ability to inhibit mitochondrial transport when treated on sensory neurons.^22^^,^^28^ Based on this finding, we sought to determine the effect of ESTIM as a neuroprotective mechanism against two different antineoplastic drugs, PTX and L-OHP. Global stimulation with chemotherapy drug treatment was initially performed to analyze the effect of ESTIM on axon length and mitochondrial trafficking, similar to the frequency response experiment. PTX is a common microtubule targeting anti-cancer drug, which in turn affects the axonal transport mechanisms leading to peripheral degeneration. From our results, 10 Hz stimulation provided the highest degree of neuroprotection against PIPN, significantly reducing the degeneration effects of PTX compared to control sample. Likewise, mitochondrial trafficking was enhanced, where 100 Hz provided the largest change in trafficking after stimulation. Based on ESTIM’s ability to enhance mitochondrial trafficking, it was speculated that if we could enhance the axonal trafficking within the cell in response to PTX treatment, it would provide neuroprotection against PIPN. Similarly, L-OHP was treated globally at a concentration of 5 μM based on *in vitro* toxicity response.^36^^,^^37^ Due to the DNA targeting mechanism of L-OHP treatment, the effects of enhanced axonal transport were expected to be less than those of PTX. L-OHP treatment targets DNA replication cycle within the nucleus, and it is assumed to likewise affect the DNA within mitochondria.^57^^,^^58^ Mitochondrial observation is key in response to L-OHP treatment as mitochondria has recently been associated with signaling responses associated with cell death.^59^ Although L-OHP targets DNA replication, ESTIM is still shown to have a neuroprotective effect on L-OHP-treated samples. Mitochondrial trafficking was also analyzed for L-OHP-treated samples to further understand how enhanced axonal transport can be used as a neuroprotective mechanism against CIPN. Although L-OHP has a different targeting mechanism than PTX, based on the results it can be seen that low-frequency stimulation still enhanced anterograde mitochondrial trafficking, where 100 Hz showed the highest increase over the stimulation period. It is important to note that mitochondrial trafficking enhancement was surprisingly similar when comparing PTX and L-OHP. Although axon length analysis showed a difference in results for each drug, mitochondrial enhancement was similar between the two, potentially indicating a similar enhancement mechanism by ESTIM. Based on our results, the axonal transport enhancement by ESTIM proved an effective neuroprotection mechanism against CIPN. Further research is needed to determine if ESTIM directly affects mitochondria trafficking as a primary or secondary mechanism. Based on this study, we speculate that the increased neural activity by low-frequency stimulation enhances axonal transport; however, the specific signaling mechanisms are still unknown at this time. Further research is needed to fully understand the signaling pathways behind neuromodulation-enhanced regeneration. Based on the results found, ESTIM has the ability to be an effective method to help patients who are experiencing painful CIPN.

In order to determine the most effective treatment method for ESTIM, axonal stimulation was performed on PTX- and L-OHP-treated samples. It was necessary to understand if there were any major differences between global and axonal stimulation since axonal stimulation is a preferred treatment method in clinical settings. Axonal stimulation allows for isolated electric fields at the region of injury, which in turn can be a safer and more effective treatment option for patients. Axonal stimulation effect against PTX- and L-OHP-induced degeneration was significant due to CIPN being a focal injury model in patients. For PTX-treated samples, 100 Hz stimulation had the highest neuroprotective effect, while for L-OHP-treated samples, 100 Hz and 1 kHz stimulation had equally the highest protection. Based on the results, axonal and global stimulation had similar effectiveness, justifying the ability to use focal stimulation as an effective method for neuroprotection against CIPN. The similarities between global and focal stimulation could be attributed to the electric field distribution within the cell. Previous studies have confirmed that single-cell stimulation can illicit increased neural activity throughout the entire cell *in vitro* and *in vivo*.^38^^,^^60^ Axonal stimulation does not have any lesser effect on neuroprotection than global and can be determined as an effective treatment method in clinical settings.

Although mitochondrial trafficking was the governing subcellular organelle that was studied, it may not be the only organelle that is affected by neuromodulation. Further research is needed to determine how other mobile organelles are affected by ESTIM. To help answer the question of enhanced axonal transport as a neuroprotective mechanism, a supplementary lysosome trafficking study was performed as a supplementary study shown in Figure S1. The effect of lysosome trafficking mirrored mitochondrial data, showing that ESTIM enhanced trafficking of lysosomes within DRGs. This supplementary data help back up the claim that ESTIM may enhance axonal trafficking as a neuroprotective mechanism against CIPN.

Based on the results of this study, mitochondrial enhancement by ESTIM is a proposed mechanism behind ESTIM-mediated neuroprotection against CIPN. Mitochondrial trafficking enhancement shows to be a key aspect of neuroprotection, which has the potential to help many patients suffering from CIPN. Based on this study, non-invasive techniques can be developed to generate electric fields, enhancing mitochondrial trafficking in clinical settings to treat CIPN or other neurodegenerative disorders or diseases.

### Limitations of the study

There were several limitations of this study. First, mitochondrial trafficking dye has an inherent diffusion rate, which may influence the trafficking analysis. Hydrostatic pressure and flow induction were performed to reduce this aspect; however, we have no way to ensure total blockage of dye diffusion at this time. Displacement over time data were manually recorded, where time-lapse images showed frame shifting, and axonal degradation in response to chemotherapy drugs, making it difficult to collect accurate velocity data from mitochondrial trafficking. At this time, we are unable to determine the signaling pathways and direct mechanism associated with ESTIM-enhanced mitochondrial transport. We hope to address all these challenges in future studies.

## STAR★Methods

### Key resources table

**Table:** 

REAGENT or RESOURCE	SOURCE	IDENTIFIER
**Chemicals, peptides, and recombinant proteins**		

Mitotracker Green FM	Invitrogen	CAT# M7514
Trypsin stable cell	Sigma aldrich	CAT# SLCB7549
Laminin	Corning	CAT# 354232
Poly-D-Lysine	EMD Millicore	CAT# A-003-E
Calcien AM	Corning	CAT# 354217
Oxaliplatin	adipoGen	CAT# AG-CR1-3592-M025
Paclitaxel	Selleck chem	CAT# 51150
Collegenase	Gibco	CAT# 17018-029
Lysosomal Stain	abcam	CAT# AB 112137
PBS	Gibco	CAT# 10010-023
Neurobasal Media	Gibco	CAT# 21103-049
L-15	Gibco	CAT# 11415-064

**Experimental models: Cell lines**		

Sprague-Dawley Rat: Dorsal Root Ganglion Neurons	Charles River, NC	

**Software and algorithms**		

PowerPoint	Microsoft	
Word	Microsoft	
Excel	Microsoft	
Lightroom	Adobe	
Prism 8	GraphPad	

### Resource availability

#### Lead contact

Further information and requests for resources and reagents should be directed to and will be fulfilled by the lead contact, Bayne Albin (Balbin@charlotte.edu).

#### Materials availability

This study did not generate new unique reagents.

#### Data and code availability

This study does not report original new code. Any additional information required to reanalyze the data reported in this paper is available from the lead contact upon request.

### Experimental model and study participants details

#### Cell culture

All animal experiments were conducted in accordance with protocols approved by the Institutional Animal Care and Use Committee (IACUC). Dorsal Root Ganglion (DRG) neurons were isolated from E−15 embryos collected from a Sprague-Dawley Rat. DRG explants were carefully collected and enzymatically dissociated using 0.25% Trypsin in L-15 medium (Sigma Aldrich). DRGs were suspended in a modified Neurobasal medium containing 1.0% Fetal Bovine Serum (FBS), 20.0% Glucose, 1.0% Penicillin/Streptomycin, B-27 supplement, 2 M _L-_glutamine, and 10 ng/mL Glial Derived Nerve Growth Factor (GDNF) (Sigma Aldrich). DRG neurons were seated into the marked somal chamber of the compartmentalized devices and left to grow for 3-5 days to allow for axons to grow through the channels into the axonal side at an adequate length.

To limit evaporation, a small cotton ball soaked in sterile distilled water and 1.0% Penicillin/Streptomycin was placed in the same Petri dish as the chambers.

### Method details

#### Compartmentalized chambers

Photolithography was used to develop a master mold which contained the microchannels for the compartmentalized chamber system. Sylgard 184 polydimethylsiloxane (PDMS) (Dow chemical company USA) was used to conduct standard soft lithography, where it was poured onto the master micro-mold followed by the removal of air bubbles by a desiccator (SP Scienceware, USA) and was allowed to cure overnight at 80°C. PDMS was removed from the master mold where two adjacent holes were punched on either side of the channels using a 6 mm diameter biopsy punch (Robbins Instruments). The dual compartmentalized chambers were then bonded to a thin glass slide (Fisher Scientific) using the Plasma Etch PE-75 Plasma Asher oxygen plasma device. After, the chamber devices were sterilized by autoclave before cell seating. Once sterilized, chambers were coated with 100μg/mL Poly-D-Lysine (PDL) (Sigma-Aldrich) and 10.0 μg/mL Laminin (Corning) and left overnight at 2°C–8°C refrigerator. Chambers were then washed thoroughly using media to prepare for cell seating. Chambers were then placed into a sterilized primary cell incubator supplying 5% CO_2_ (Binder C-150 UL) to accustom for the cell culture environment.

#### Drug treatment and mitochondria staining

PTX and L-OHP were prepared as 1mM stock solutions prior to making the working solution and stored at −20°C freezer. PTX (Selleck Chemicals) was dissolved in ethanol, and L-OHP (Sigma) was dissolved in 0.9% sodium chloride. Stock solutions were further diluted using Neurobasal media. 5μM was used for both drugs as they show proper dying back neuropathy and similar neurotoxicity as in *in vivo* situations. Drugs were administered to the compartments by removing 75% of existing media and readministered with the drug containing media at 37°C for 1 h prior to fluorescent staining. Cells were stained using Calcien AM (ThermoFisher) and allowed to incubate for 30 min to 1 h after initial drug treatment. DRG’s were imaged using a Leica DMi8 Thunder Fluorescent Microscope immediately after drug treatment, and likewise 24, and 48 h after imaging. In order to image mitochondria, MitoView Green was obtained from Biotium, USA and was added to the cells for 30 min to 1 h after drug treatment, similar to the previous protocol. Media was replaced twice to reduce any background fluorescence, and immediately imaged. The compartmentalized system was covered in aluminum foil to prevent any light degradation.

Axon length measurements were performed using a glass bottom non-compartmentalized cell culture array, where embryonic DRGs were cultured for 24 h prior to CIPN drug treatment. PTX was treated at a concentration of 5 μM and administered globally 1 h prior to stimulation for all experiments. Due to the Mitochondrial trafficking effects, we previously studied with FA, it was decided to use a similar toxicity concentration for each drug to understand the neuroprotective mechanism of ESTIM against CIPN.

#### ESTIM setup

Pulse wave signal generators (Siglent) were used to create the proper square wave signal needed for proper stimulation. A constant 3 V power source was used to create a biphasic rectangular pulse lasting 0.5s every 2s with a pulse width of 0.2 ms. Platinum wires were attached to the cell culture system’s lid, where wires were attached to the signal generators shown in the schematic. The platinum wires were placed into the wells, where one electrode was placed in each well pair for global stimulation, and both electrodes were placed into the axonal chamber for focal stimulation. Control wells were not connected to the stimulation device.

#### Data analysis

Data was collected using Fiji-ImageJ to measure axon lengths in triplicate samples. Microsoft Office Excel 2022 was used to compile all measurement data, where average and standard deviation was calculated. Axon integrity was found by calculating percent difference compared to the average control (0h) for each sample. Differences in percent change were used to analyze the effect of site-specific treatment. Mitochondrial analysis used a similar approach, where mitochondrial number was compared against the control (0h), normalizing the dataset. Data was presented as a percentage of the control value, showing a positive or negative change in comparison.

To quantify the mitochondrial displacement, images were analyzed with the aid of ‘MTrackJ’ plugin for manual tracking of individual mitochondria across the frames until the mitochondria disappears from view, stops moving, or moves out of frame. 30-min timelapse imaging was preformed using the built-in Leica software. Images were taken at 1-min intervals for 30 min. Manual displacement was calculated by measuring the distance traveled of a singular mitochondrion along the thirty frames.

### Quantification and statistical analysis

All datasets were presented as a mean ± standard deviation except where noted. Datasets were grouped as triplicate N = 3. The probability (p*-*value) between groups was analyzed by the two-tailed *t*-test provided in Microsoft Office Excel 2022 unless otherwise stated. p-value less than 0.05 was considered statistically significant.

## Supplemental information

Figure S1. Lysosomal trafficking response to electrical stimulation frequency range Lysosome trafficking was determined using the same experimental methodologies as ESTIM. Using a lysosomal staining kit, trafficking response was determined using the compartmentalized chamber. Stimulation was performed at 10 Hz, 100 Hz, 1 kHz, and 100 kHz to analyze the trafficking response similar to Figure 2F. Results found that low-frequency stimulation enhanced lysosomal trafficking similar to mitochondria.
