# Supplementary material for: Electrical stimulation enhances mitochondrial trafficking as a neuroprotective mechanism against chemotherapy-induced peripheral neuropathy
Source: iScience. 2024 Jan 30;27(3):109052. doi: 10.1016/j.isci.2024.109052 (PMC10875116; doi:10.1016/j.isci.2024.109052)
Supplement: Figure S1. Lysosomal trafficking response to electrical stimulation frequency range — Lysosome trafficking was determined using the same experimental methodologies as ESTIM. Using a lysosomal staining kit, trafficking response was determined using the compartmentalized chamber. Stimulation was performed at 10 Hz, 100 Hz, 1 kHz, and 100 kHz to analyze the trafficking response similar to Figure 2F. Results found that low-frequency stimulation enhanced lysosomal trafficking similar to mitochondria. [file mmc1.pdf]

iScience, Volume *27*

Supplemental information

Electrical stimulation enhances mitochondrial trafficking as a neuroprotective mechanism against chemotherapy-induced peripheral neuropathy

Bayne Albin, Prashant Adhikari, Arjun Prasad Tiwari, Khayzaran Qubbaj, and In Hong Yang

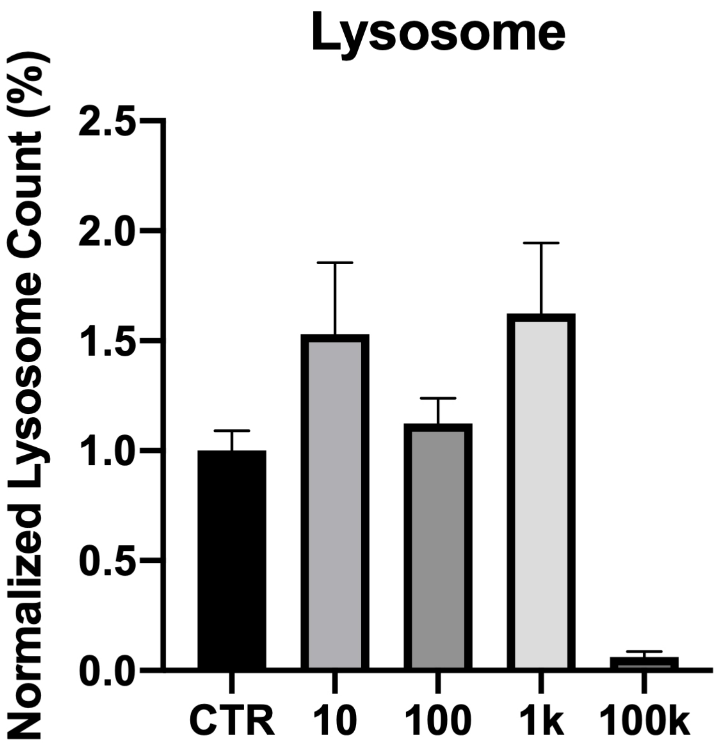

Lysosome
Normalized Lysosome Count (%)
2.5
2.0
1.5
1.0
0.5
0.0
CTR
10
100
1k
100k
